# Functional neurogenomic responses to acoustic threats, including a heterospecific referential alarm call and its referent, in the auditory forebrain of red-winged blackbirds

**DOI:** 10.1038/s41598-024-51797-y

**Published:** 2024-01-25

**Authors:** N. D. Antonson, J. K. Enos, S. L. Lawson, F. M. K. Uy, S. A. Gill, K. S. Lynch, M. E. Hauber

**Affiliations:** 1 Department of Evolution, Ecology, and Behavior, School of Integrative Biology, University of Illinois, Urbana-Champaign, IL USA; 2 Department of Ecology, Evolution, and Organismal Biology, Brown University, Providence, RI 02912 USA; 3 Illinois Natural History Survey, Prairie Research Institute, University of Illinois, Urbana-Champaign, IL USA; 4 Carl R. Woese Institute for Genomic Biology, University of Illinois, Urbana-Champaign, IL USA; 5 Department of Biology, University of Rochester, Rochester, NY USA; 6 Department of Biological Sciences, Western Michigan University, Kalamazoo, MI USA; 7 Department of Biology, Hofstra University, Hempstead, NY USA; 8 Advanced Science Research Center and Program in Psychology, Graduate Center of the City University of New York, New York, NY USA

**Keywords:** Auditory system, Ecology

## Abstract

In animal communication, functionally referential alarm calls elicit the same behavioral responses as their referents, despite their typically distinct bioacoustic traits. Yet the auditory forebrain in at least one songbird species, the black-capped chickadee *Poecile atricapillus*, responds similarly to threat calls and their referent predatory owl calls, as assessed by immediate early gene responses in the secondary auditory forebrain nuclei. Whether and where in the brain such perceptual and cognitive equivalence is processed remains to be understood in most other avian systems. Here, we studied the functional neurogenomic (non-) equivalence of acoustic threat stimuli perception by the red-winged blackbird *Agelaius phoeniceus* in response to the actual calls of the obligate brood parasitic brown-headed cowbird *Molothrus ater* and the referential anti-parasitic alarm calls of the yellow warbler *Setophaga petechia,* upon which the blackbird is known to eavesdrop. Using RNA-sequencing from neural tissue in the auditory lobule (primary and secondary auditory nuclei combined), in contrast to previous findings, we found significant differences in the gene expression profiles of both an immediate early gene, ZENK (egr-1), and other song-system relevant gene-products in blackbirds responding to cowbird vs. warbler calls. In turn, direct cues of threats (including conspecific intruder calls and nest-predator calls) elicited higher ZENK and other differential gene expression patterns compared to harmless heterospecific calls. These patterns are consistent with a perceptual non-equivalence in the auditory forebrain of adult male red-winged blackbirds in response to referential calls and the calls of their referents.

## Introduction

In animal communication, signalers use functionally referential calls to denote objects or events in the environment, to which listeners respond specifically, as influenced by context and relevance^1^. Receivers of referential calls are not always conspecifics^2^, and in many systems anti-predatory alarm calls are eavesdropped upon by heterospecific listeners in sympatry with the calling species (e.g.^3,4^). Amongst hosts of obligate brood parasitic species, which lay their eggs into the nests of other species, the functionally referential alarm calls of the yellow warbler *Setophaga petechia* (hereafter: warbler)^5^ are produced in response to the brood parasitic brown-headed cowbird *Molothrus ater* (hereafter: cowbird). In turn, these calls are intercepted by and elicit mobbing responses in another host species of the cowbird, the red-winged blackbird *Agelaius phoeniceus* (hereafter: blackbird)^6^. Specifically, female and male blackbirds respond aggressively to both the warblers’ anti-parasitic seet calls and the cowbird females’ chatter calls, but do so only during the laying and incubation stages of the blackbird reproductive cycle when cowbirds parasitize nests, and not during the nestling stage when the broods are safe from cowbird parasitism^7^.

The neural processing of functional referential calls by eavesdropping heterospecifics remains unclear. Prior work on the neural basis of functionally referential vocalizations in songbirds is limited to conspecific studies on black-capped chickadees *Poecile atricapillus* (hereafter: chickadee) (e.g.^8^, a species that produces graded alarm calls in response to different predatory owl calls of varying threat intensities^9^. Accordingly, in response to playbacks of conspecific alarm calls or calls of the referents, similar activation of the immediate early gene (IEG) ZENK (also called egr-1^10^), occurred in the secondary auditory forebrain areas of the caudomedial mesopallium (CMM) and caudomedial nidopallium (NCM^11^). The need for a targeted neurogenomic focus on these two specific forebrain nuclei is implied by many earlier songbird IEG studies that have shown differentially expressed IEGs in CMM and NCM in response to conspecific vs. heterospecific songs and familiar vs. unfamiliar calls (e.g.^12^).

No studies that we know of have yet addressed the neural basis of heterospecific eavesdropping on functionally referential alarm calls. Therefore, we designed a playback experiment on recently captured wild adult male blackbirds to assay their rapid neurogenomic responses to warbler seet calls and the calls of their referent, the cowbird. Based on our prior behavioral data collected in the field from wild blackbirds (i.e.^6,13^), we predicted that the auditory forebrain of blackbirds will show similar levels of IEG activation responses to warbler seet calls and cowbird chatter calls, and also that responses will be similar to calls of other biologically meaningful direct threats (e.g., a nest predator, a conspecific intruder) relative to the calls of a harmless heterospecific.

Instead of the immuno-histochemistry approach utilized in Avey et al.^11^, we used an RNA-sequencing approach (sensu^14^) in our experiment to measure neural activity in the auditory lobule of the songbird forebrain, which is inclusive of field L, CMM and NCM (sensu^15^). Unlike CMM and NCM, field L does not always stain for and show differential ZENK expression levels to differently salient acoustic stimuli in immuno-histochemistry experiments (e.g.^11^), but it can be selective for conspecific over heterospecific vocalizations when measured via neurophysiological single-unit spike rates (e.g.^16^). Therefore, we obtained RNA-sequencing data from the auditory lobule and conducted both candidate gene and weighted gene correlation network analyses. Contrasting social experiences (e.g.^17^), including various acoustic playbacks to icterid birds, rapidly (after just 30 min. of exposure, followed by immediate RNA-collection) activate differential gene expressions patterns in both the brain^18^ and the periphery (blood^13^), respectively. As such, we exposed our subjects to this same length of stimulus presentation, followed by immediate brain tissue collection. This was also different between Avey et al.^11^ and our work (here), as their IEG techniques required a 60 min. dark, non-exposure period following the 30 min. playback period^11^ (discussed below).

## Material and methods

### Field work

We captured wild adult male blackbirds (n = 22) during their breeding season (April–July^19^ in 2019 and 2020 at two USA localities (Champaign County, IL, and Queens, NY). All experimental licenses and protocols were approved by the Institutional Animal Care and Use Committee of the University of Illinois at Urbana-Champaign (#18040), adhered to relevant state and federal regulations, and are reported following ARRIVE guidelines.

Birds in IL were captured at the Phillips Tract Natural Area in grain-bated Potter-traps, which were set up in blackbird breeding habitat throughout the season. Once captured, the subjects were transported to the University of Illinois Urbana-Champaign campus and moved to a sound-proof chamber for 30 min. of habituation to the playback experimental set-up. During this time they were provided with light and *ad lib* food and water. Birds in NY were captured by the US Department of Agriculture at the John F. Kennedy Airport and transported to Hofstra University. Subjects were exposed to auditory stimuli on the IL and NY campuses following the same procedures as described below. Preliminary comparisons revealed no effect of capture site on gene-expression patterns (F1 = 0.45, p = 0.51) and so site was not included in subsequent statistical models.

### Playback experiment

In the laboratory, after habituation, we turned off the light in the chamber and began a 30-min. playback sequence (see below) of one the five stimulus types: conspecific calls (male blackbird chatters; n = 5 subjects), parasitic calls (female cowbird chatters; n = 5 subjects), referential alarm calls (warbler seets; n = 4 subjects), nest predator calls (vocalizing blue jays *Cyanocitta cristata*; n = 4 subjects), and harmless sympatric calls (negative control: mourning dove coos *Zenaida macroura*; n = 4 subjects). Representative spectrograms of these acoustically distinct calls have been published elsewhere by us^6,13^. These were the final sample sizes after filtering out data from birds that were collected outside of the breeding season. We did not collect robust behavioral data, such as vocal responses of the subjects during the full course of the sound presentations, but anecdotally we never heard the blackbirds calling at the onset of the playbacks. Once a playback treatment was completed, we euthanized the subject with an overdose of isoflurane, followed by rapid decapitation. The brain was exposed, laterally bisected, and the left auditory lobule was identified visually along the brain’s central plane, excised within 10 min after euthanasia, flash-frozen on dry ice, then stored until RNA-extraction at − 80 °C (following^18^ for a related icterid species, the brown-headed cowbird). RNAse-away (Thermo Fisher Scientific, Waltham, MA) was used on all surfaces and instruments to minimize RNA degradation.

Playback sequences consisted of publicly sourced songs or calls (from the online database: Xeno-Canto and from^6^). For each playback file, we selected a unique song or call from one individual within our source recordings, that alternated with 10 s of silence in between them, for 10 min. We then inserted 10-min silence, after which we continued with the 10-min sound playback again. This design mirrored our playback paradigm used for a peripheral (blood) RNA-sequencing study on wild breeding male blackbirds in the field^13^. We constructed two different files (i.e., exemplars A or B, see Figshare.com), each with a different individual’s vocalizations, for each playback type to reduce pseudoreplication of stimulus-presentations^20^. Each sound file was 100–10,000 Hz bandpass filtered and standardized at peak amplitude 25 kU in Audacity 2.2.0. Playbacks were conducted at 65 dB when measured at 0.5 m from the speaker. We do not provide a comparative bioacoustic analysis of each playback stimulus type, as they represent natural exemplars of different species’ calls, thereby varying in most acoustic features that we might select to compare and contrast.

### RNA-extraction and -sequencing

RNA was extracted from each tissue sample and DNase purified (following^18^). RNA quality was assessed for degradation using a Bioanalyzer (Agilent, Wilmington, Delaware, USA) and all samples met the appropriate integrity score (RIN > 7.0). All library preparation and sequencing were performed at the University of Illinois at Urbana-Champaign Roy J. Carver Biotechnology Center. The libraries were pooled, quantitated by qPCR, and sequenced on one lane of an Illumina NovaSeq, producing 30–40 M paired-end 150 bp reads.

Illumina adapters were removed using Trimmomatic v0.39. We then aligned reads to the red-winged blackbird reference genome and annotation file (NCBI Accession: GCA_013398535.1_ASM1339853v1) using STAR v. 2.7.6a. Both the STAR index generation and STAR alignment were performed using default settings detailed in the program manual. Read counts were generated for genes using the featureCounts command in the Subread program (v. 2.0.0).

### Statistical analyses

Our data are available open access at Figshare.com and through NCBI (see below). To compare our data with those of the published literature, we first extracted normalized gene counts with a variance stabilizing transformation, from the RNA-sequencing data for a focal IEG, ZENK, as it was assayed in the prior chickadee referential alarm calling study^11^. We then conducted an uncorrected ANOVA on the expression levels of ZENK across all of the stimulus types, and used Fisher’s least significant difference (LSD) post-hoc tests to calculate pairwise differences. The full model’s residuals were normally distributed (Shapiro–Wilk’s test, W = 0.97, p = 0.60), justifying our use of parametric tests. This ANOVA and the Fisher’s LSD post-hoc tests were conducted using the DescTools package in R 4.2.2^21^. We set alpha = 0.05. Because our sample sizes were relatively small (see perspective by^14^ but also^18^), we focus our conclusions and discussion below based on the sufficiently powered statistically significant patterns rather than the potentially underpowered statistically non-significant differences^22^.

Transcriptome-wide differential gene expression analysis was also performed in R using DESeq2^23^. Raw counts were filtered to only analyze genes where all samples included a minimum gene count of 10 to filter out lowly expressed transcripts. Counts were normalized using the median of ratios method. Contrasts were then defined as comparisons between individual treatment groups and mourning dove coo which served as a positive auditory control. Post-hoc tests adjusted for false discovery rate using Benjamini–Hochberg corrections for multiple hypothesis testing were used to assign p-values (alpha = 0.05). A Venn diagram of overlapping DEGs between treatments and the negative control (mourning dove coo) was generated using Venny 2.1 (https://bioinfogp.cnb.csic.es/tools/venny/index.html). Gene ontology enrichment analysis was performed using statistical overrepresentation tests on PantherDB (http://www.pantherdb.org/) with an FDR correction for multiple hypothesis testing using the orthologous human genes to those found differentially expressed in our dataset. The GO SLIM Biological Process annotation was used to assign ontology.

Weighted gene correlation network analysis was then performed to determine coexpression of genes in response to auditory stimulus using the WGCNA package in R^24^. A soft threshold of 14 was used for network construction which represented the plateau of mean connectivity. We generated a signed network with a minimum module size of 40 genes and a merge cut height of 0.20. Once we determined coexpression modules, we screened the genes in each module for known candidate song markers that might be relevant to auditory threat reception using information from http://www.zebrafinchatlas.org/.

## Results

### ZENK expression pattern

**Figure 1:**
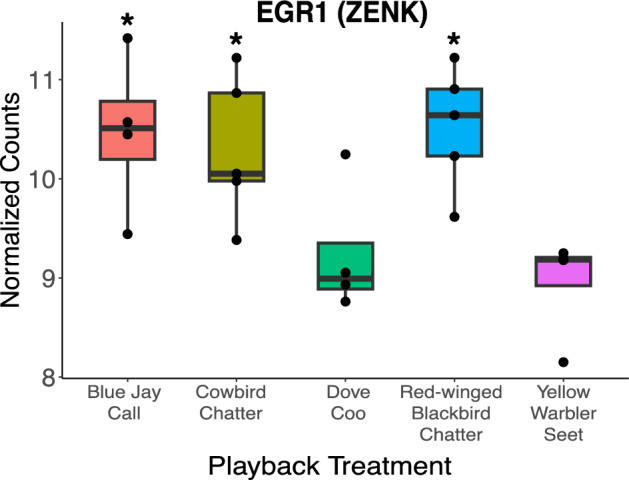
ZENK (egr-1) expression levels (counts) in the auditory lobule of adult red-winged blackbird males in response to different playback stimulus types, including anti-parasitic alarm calls of the yellow warbler (referential alarm), male blackbird calls (conspecific), mourning dove calls (control), brown-headed cowbird chatter calls (brood parasite) and blue jay calls (nest predator). Whiskers and boxplots indicate the 10th, 25th, 50th, 75th, and 90th percentile of the data spread (black dots). The “*” denotes significant differences compared to both the control dove coos and experimental yellow warbler seets adjusted for multiple comparisons using the Benjamini–Hochberg correction.

We detected an experiment-wide effect of the playback treatment types in our normalized ZENK gene count data from the auditory lobule of blackbirds (F_4,17_ = 5.032, p = 0.00733; Fig. 1). Post-hoc adjusted comparisons with Fisher’s LSD revealed significant posthoc differences between cowbird chatter calls and referential warbler seet calls (corrected p = 0.020), blackbird chatters and warbler seets (p = 0.019), and between blue jay calls and warbler seets (p = 0.019). Compared to the negative control of morning dove, cowbird (p = 0.040), blackbird (p = 0.022), and blue jay (p = 0.030) calls all had significantly higher ZENK expression. Dove coos and yellow warbler seets treatments were not significantly different from one another in their ZENK expression levels (p = 0.534).

### Differential gene expressions

When comparing our four treatment stimuli to our negative control (dove coos), the warbler referential alarm calls did not generate any differentially expressed genes (DEGs). However, when the negative control was compared to the other three treatment groups each generated differentially expression gene sets with parasitic cowbird chatters having 484 DEGs, conspecific blackbird chatters having 251 DEGs, and predatory blue jay calls having 642 DEGs (Supplementary Fig. 1a). Of the three treatments producing DEGs, 155 overlapping genes were differentially expressed across all of these treatments, while fewer were shared in each of the pairwise comparisons (Supplementary Fig. 1a,b). Blue jay calls represented the treatment with the largest number of unique differentially expressed genes. When gene ontology over-representation analysis was performed, only the comparisons between all three direct threat cues with overlapping DEGs, and the comparison between cowbird-control and blue jay-control generated over-represented GO terms (Supplementary Fig. 1b,c). The differentially expressed genes for the comparison between all 3 direct threat cues represented ontologies of neurotransmitter regulation, while those DEGs associated specifically with the overlap of cowbird-control and blue jay-control comparisons represented ontologies of transcriptional regulation.

### Weighted gene coexpression network analysis

**Figure 2:**
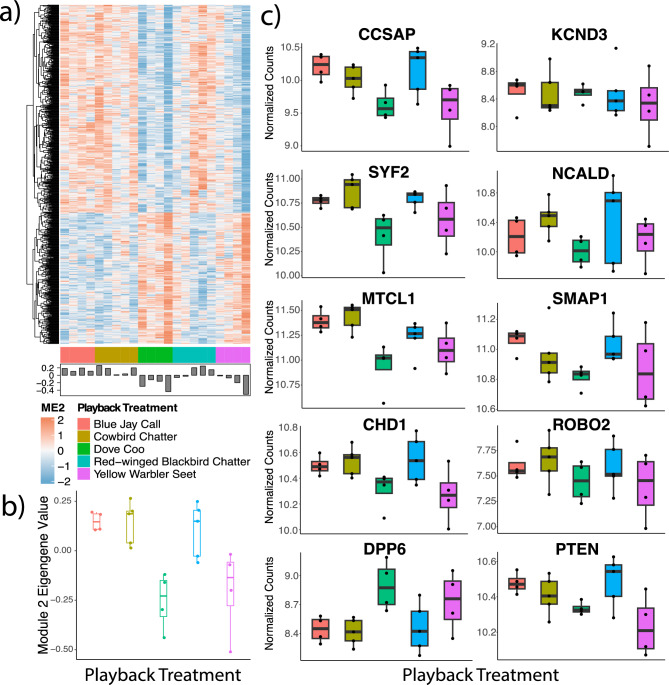
(**a**) Module 2 of the weighted gene coexpression network analysis demonstrated similar trends to the a priori ZENK expression analysis with (**b**) eigengene values being higher in response conspecific, parasite, and predator calls and lower in response to the negative controls and heterospecific alarms. Whiskers and boxplots indicate the 10th, 25th, 50th, 75th, and 90th percentile of the respective data (black dots). (**c**) Boxplots of genes from Module 2 that are considered markers of the song system according to the Zebra Finch Expression Brain Atlas (http://www.zebrafinchatlas.org/).

WGCNA was then performed, and only Module 2 had a significant correlation with the treatments (F = 3.991, adjusted p = 0.049; Fig. 2a). These results, summarized by the eigengene values, paralleled the pattern of the ad hoc EGR1 (ZENK) analysis (F_4_ = 8.223, p < 0.001; Fig. 2b), with the dove coos eigengene value being significantly lower than jay calls (p < 0.001), cowbird chatters (p < 0.001), and blackbird chatters (p < 0.002), and the warbler seets showing a similar pattern of an eigengene value being significantly lower than the same treatments of jay (p = 0.003), cowbird (p = 0.002), and blackbird (p = 0.005) calls. There was no significant difference in eigengene value between warbler seets and dove coos (p = 0.595). Module 2 contained 806 coexpressed genes, including 59 transcription factors and 10 known song markers (including transcription factors CHD1 and PTEN phosphatase). Gene ontology overrepresentation analysis showed that genes in module two were broadly associated with 2 groups of GO Terms as well as other miscellany, summarized as transcription related genes and those related to signal transduction and neurotransmitter secretion (Supplementary Fig. 2).

### Song marker candidate genes and network connectivity

From our screen of the 806 genes comprising Module 2 of our WGCNA, we identified 10 genes that are known markers of the avian song system within the songbird brain (Fig. 2c). Although the auditory system (including the lobule) is not a constituent component of the oscine song system, the latter receives extensive auditory input from the former (e.g.^25^), that might also imply functional genomic connectivity between the two. Of these 10 genes, 6 had significant ANOVA p-values (Table S1). We then used Fisher’s LSD post hoc tests to compare blackbird chatters, cowbird chatters, and jay calls to warbler seets and dove coos. Finally, we followed up our candidate post hoc comparisons by analyzing the connectivity of these song markers within Module 2 (Table 1). CCSAP was the most connected song marker and had significantly different expression patterns in response to blackbird chatters and jay calls compared to both warbler seets and dove coos (Tables 1 and S1). SYF2 factor was the 2nd most connected song marker and was significantly different between dove coo when compared to cowbird chatters, blackbird chatters, and jay calls, but was only significantly different between warbler seets and blackbird chatters (Tables 1 and S1). MTCL1 was significantly differentially expressed amongst all treatment comparisons to dove coos and warbler seets, with the exception of blackbird chatters and warbler seest, and was the 3rd most connected song marker (Tables 1 and S1). CHD1 ranked 4th and was significantly different when comparing warbler seets and dove coos to cowbird and blackbird chatters (Tables 1 and S1). DPP6 had no significant post hoc comparisons to warbler seets, and PTEN phosphatase had no significant post hoc comparisons to dove coos, while KCND3, NCALD, SMAP1, and ROBO2 had no significant post hoc comparisons at all. It is noteworthy that no song marker genes produced significant post hoc comparisons between warbler seets and dove coos (Table 1 and S1). Several transcription factors ranked highly in the network connectivity analysis of module 2, particularly SNW1 (Table 1).

**Table 1:** Network connectivity metrics for module 2. Genes in italics represent the top 5 transcription factors in the module and the 10 genes in bold represent known song circuit markers from http://www.zebrafinchatlas.org. PTEN phosphatase and CHD1 are both bold and italicized as they are both known song markers and transcription factors.

Rank by degree	Gene	Degree	Closeness centrality	Edge betweenness	Hub score
1	ROA2	290	0.002	37,111.538	1
*2*	*SNW1*	*281*	*0.002*	*35,440.149*	*0.969*
3	HAP28	277	0.002	35,994.349	0.975
4	TCRG1	258	0.002	24,155.926	0.983
5	ZN362	220	0.001	18,209.899	0.898
*20*	*SETD2*	*76*	*0.001*	*1600.550*	*0.438*
*29*	*ADNP*	*54*	*0.001*	*979.850*	*0.349*
*75*	*REST*	*20*	*0.001*	*13.962*	*0.222*
*76*	*EPC2*	*20*	*0.001*	*909.712*	*0.217*
131	**CCSAP**	**15**	**0.001**	**12.348**	**0.166**
164	**SYF2**	**12**	**0.001**	**0.000**	**0.163**
230	**MTCL1**	**8**	**0.001**	**0.466**	**0.098**
*259*	***CHD1***	***7***	***0.001***	***0***	***0.073***
310	**DPP6**	**5**	**0.001**	**0**	**0.046**
462	**KCND3**	**2**	**0.001**	**0**	**4.81E−05**
485	**NCALD**	**2**	**0.067**	**0**	**2.77E−18**
627	**SMAP1**	**1**	**NA**	**0**	**2.31E−18**
676	**ROBO2**	**1**	**NA**	**0**	**2.31E−18**
*742*	***PTEN***	***1***	***NA***	***0***	***2.31E*−*18***
802	ZN638	1	NA	0	2.31E−18
803	LMTK3	1	NA	0	2.31E−18
804	TIMP4	1	NA	0	2.31E−18
805	JMJD8	1	NA	0	2.31E−18
806	ETV3	1	NA	0	2.31E−18

## Discussion

Salient auditory stimuli, including direct cues of conspecific intruders (male blackbird chatters) and nest predators (blue jay calls) elicited significantly greater ZENK and overall gene activation patterns in the auditory lobule of male red-winged blackbirds in the breeding season, compared to our positive auditory control (harmless dove calls). Responses to cowbird chatters (484 DEGs), blackbird chatters (251 DEGs), and blue jay calls (642 DEGs) also differed substantially regarding differential gene expression from the negative control dove coos whereas warbler seets did not. This implies that relatively short exposure (30 min. followed by immediate tissue collection) to acoustic threats can generate relatively rapid differences in gene expression levels between some threatening and non-threatening stimuli. Thus, contrary to our prediction, the auditory lobule of male blackbirds appears to differentially process the anti-parasitic referential alarm calls of yellow warblers and the referent brood parasitic brown-headed cowbirds’ chatters. There are several potential explanations for this latter pattern and we discuss them in turn.

First, the auditory lobule includes both the primary auditory nucleus of field L and the secondary nuclei of CMM and NCM. In contrast, the prior study on black-capped chickadees only assessed IEG expression in the secondary auditory forebrain nuclei of CMM and NCM^10^. Field L is known for its strong species-specific electrophysiological responses between conspecific and heterospecific stimuli (e.g.^16^), whereas CMM and NCM are (also) involved in more fine-tuned, familiarity-based discrimination (e.g.^26^). This suggests that our RNA-sequencing data may have been dominated by field L gene expression patterns relative to CMM and NCM in the pooled tissues’ data set. In this scenario, our data do not necessarily contradict the patterns found in chickadees and future studies should focus on RNA-sequencing of the CMM and NCM nuclei (separately), perhaps using tissue punches from sectioned brain slices for anatomical accuracy (e.g.^27^). However, contrary to this scenario, prior ZENK-based analyses did not detect substantial or differential expression in those parts of field L that do express ZENK in response to different classes of salient auditory stimuli (e.g.^28^), implying that most of our ZENK mRNA counts would have come from CMM and NCM, making our study more directly comparable with the chickadee ZENK study^10^.

Second, it is possible the primary and secondary auditory forebrain regions of blackbirds do process warbler seet calls and cowbird chatter calls as distinct auditory signals, and the cognitive equivalence of these two calls arises as the signals move downstream further along the auditory pathway in the songbird forebrain. For example, the nucleus taeniae of the avian amygdala has inputs from auditory pathways, and is known to respond to more salient social stimuli with greater IEG activity relative to controls^29^, as if assigning valence to these stimulus inputs.

Third, our brain tissue-collection was preceded only by 30 min. playback directly, without a follow-up dark-period of 60 min in silence (as was done by^11^). It is possible that the differential time table for tissue harvesting yielded the different results seen between the chickadee and the blackbird data sets. For example, Uy et al.^30^ demonstrated in a social wasp species that using RNA-sequencing, a greater number of differentially expressed genes can be detected when harvesting tissue 4 h vs. immediately following the social stimulation period. Our experimental protocol did not allow us to test this latter alternative.

Fourth, our behavioral work on blackbirds’ eavesdropping on warbler alarm calls has shown that reproductive stage and context are paramount for treating warbler seet calls as salient threat stimuli. Blackbird males and females only respond aggressively to experimental warbler and cowbird calls during laying and incubation stages of the reproductive cycle, and not during the nestling stage^6,7^. Here, even though all blackbirds were caught during the local reproductive season of the species, we did not know the exact breeding stage of the males’ nests (if any), therefore, the detected neurogenomic responses may have reflected those of males across all breeding stages, rather than the specific stages when cowbirds threaten nests.

Finally, the sampled blackbirds may have varied in their exposure to yellow warbler calls prior to collection. Blackbirds are less likely to approach (and, perhaps, eavesdrop on) playbacks of seet calls when breeding further away from the nearest warbler territory^6^. Given that we did not know the nesting locations of the trapped blackbirds in relation to nearby yellow warbler territories, we cannot assess whether our subjects had abundant or limited individual or social familiarity (sensu^31,32^) with warbler referential seet calls. Because some nuclei in the auditory lobule (i.e., NCM and CMM) are known to differentially respond to familiar vs. unfamiliar vocalizations using IEG markers (e.g.^12,26,33^), it is possible that the male subjects in our sample were simply unfamiliar with warbler seet calls, thus responding to them similarly to the negative control (dove coos).

Despite the absence of specialized neurogenomic responses to seet calls, our results demonstrate greater IEG expression patterns in response to the three classes of direct threat stimuli. Blackbird males showed significantly greater responses to blackbird chatters (i.e., conspecific intruders) and heterospecific blue jay calls (nest predators) over the harmless heterospecific mourning dove coos (control playback). These heightened neurogenomic responses parallel previous behavioral work on wild blackbird males that were observed and caught on their territories, where they showed both greater behavioral responses and differential peripheral (blood) gene expression patterns in response to call playbacks representing conspecific male intruders over dove controls^13^.

Gene network connectivity also differed among threat, referent, and control stimuli. Due to the size of the module produced by the WGCNA, we focused the network connectivity analysis on known song markers that are likely to be differentially expressed in the auditory and song circuits. Of the song markers in Module 2, CCSAP had the highest network connectivity and showed graded differential expression with the highest expression in response to direct threats (red-winged blackbird chatters and blue jay calls), but no statistical difference between cowbird chatter and warbler seet. CCSAP is a song marker that causes cell proliferation and is known to vary across brain regions and in response to singing^34^. SYF2 had a wider-encompassing threat response with responses to all threat cues compared to dove coo (except warbler seets). SYF2 is upregulated in the HVC, a target of field L, the NCM, and CMM, and is an essential splice factor for cell proliferation^35^. Interestingly, PTEN has previously been implicated as a candidate gene for language readiness^36^, and was only differentially expressed compared to warbler seets and not dove coos. However, none of the song marker candidates showed expression profiles where seets and cowbird chatters were significantly different from all other treatments, providing additional evidence that the two vocalizations are not encoded as functionally equivalent in the auditory lobule.

Future work should address the gene-expression patterns of specific auditory and downstream forebrain areas response to warbler referential calls and the referent cowbird calls in blackbird males of known reproductive stages. In addition, the time course of these future experiments should allow for longer delays until brain processing, to allow neurogenomic transcription to progress further than that allowed by our 30 min. playback paradigm used for this study.

### Supplementary Information

Supplementary Information.

## Data Availability

The data used for these analyses are available publicly at https://figshare.com/s/5df86c1cc46117e5194c. Raw RNAseq reads are available through NCBI in Bioproject PRJNA1051028 (https://www.ncbi.nlm.nih.gov/bioproject/?term=1051028).
