# Supplementary material for: Functional neurogenomic responses to acoustic threats, including a heterospecific referential alarm call and its referent, in the auditory forebrain of red-winged blackbirds
Source: Sci Rep. 2024 Jan 25;14:2155. doi: 10.1038/s41598-024-51797-y (PMC10810909; doi:10.1038/s41598-024-51797-y)
Supplement: Supplementary file 1 — Supplementary Information. [file 41598_2024_51797_MOESM1_ESM.docx]

**Functional neurogenomic responses to acoustic threats, including a heterospecific referential alarm call and its referent, in the auditory forebrain of red-winged blackbirds**

Antonson ND, Enos JK, Lawson SL Uy FMK, Gill SA, Lynch KS, Hauber ME

*Supplementary Information*

*Supplemental Table 1. ANOVA results and Benjamini-Hochberg post hoc comparisons of treatments compared to dove coo and yellow warbler seet for candidate song markers. Comparisons with no significant differences marked as N.S. except the direct comparison between dove coo and warbler seet.*


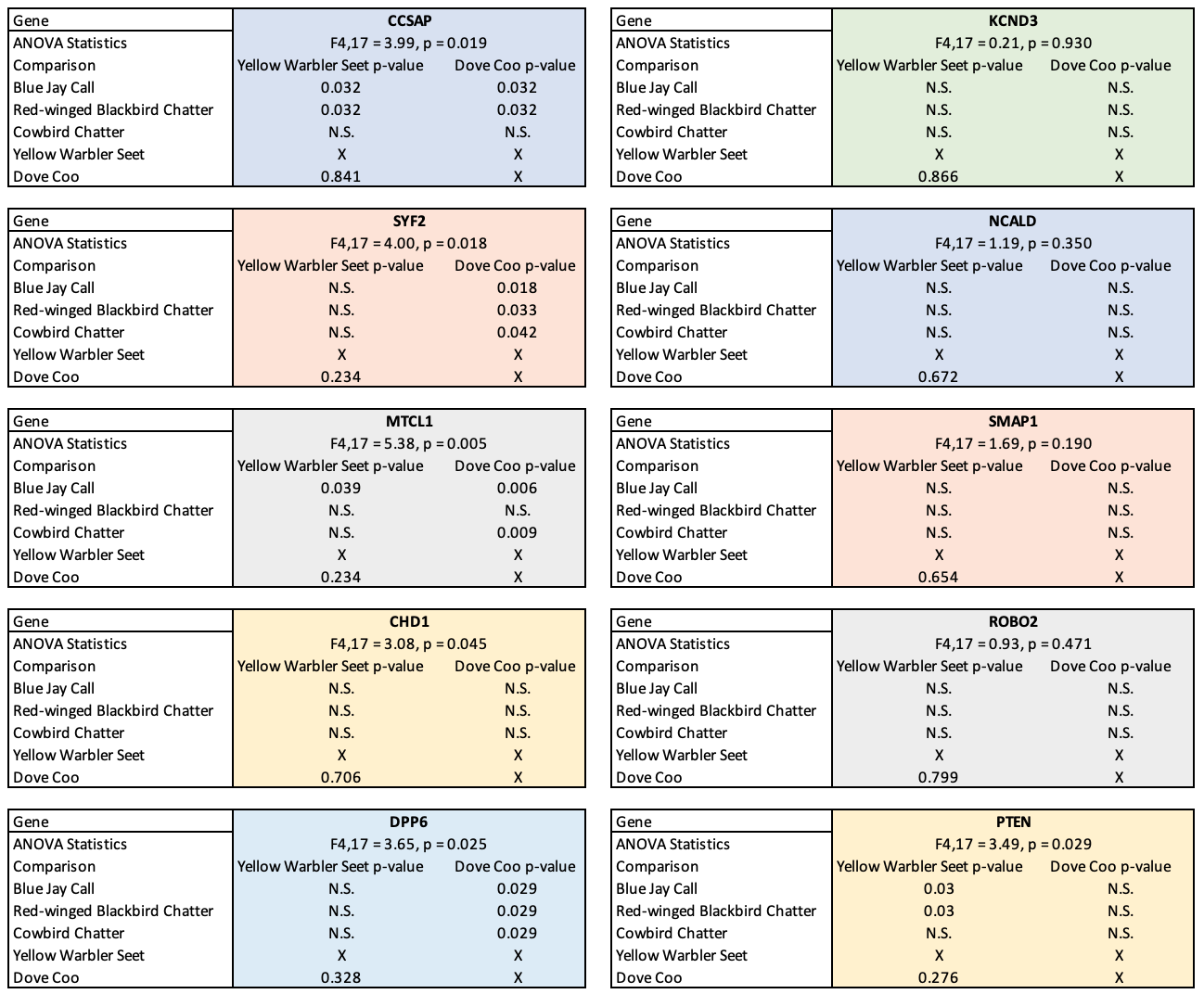


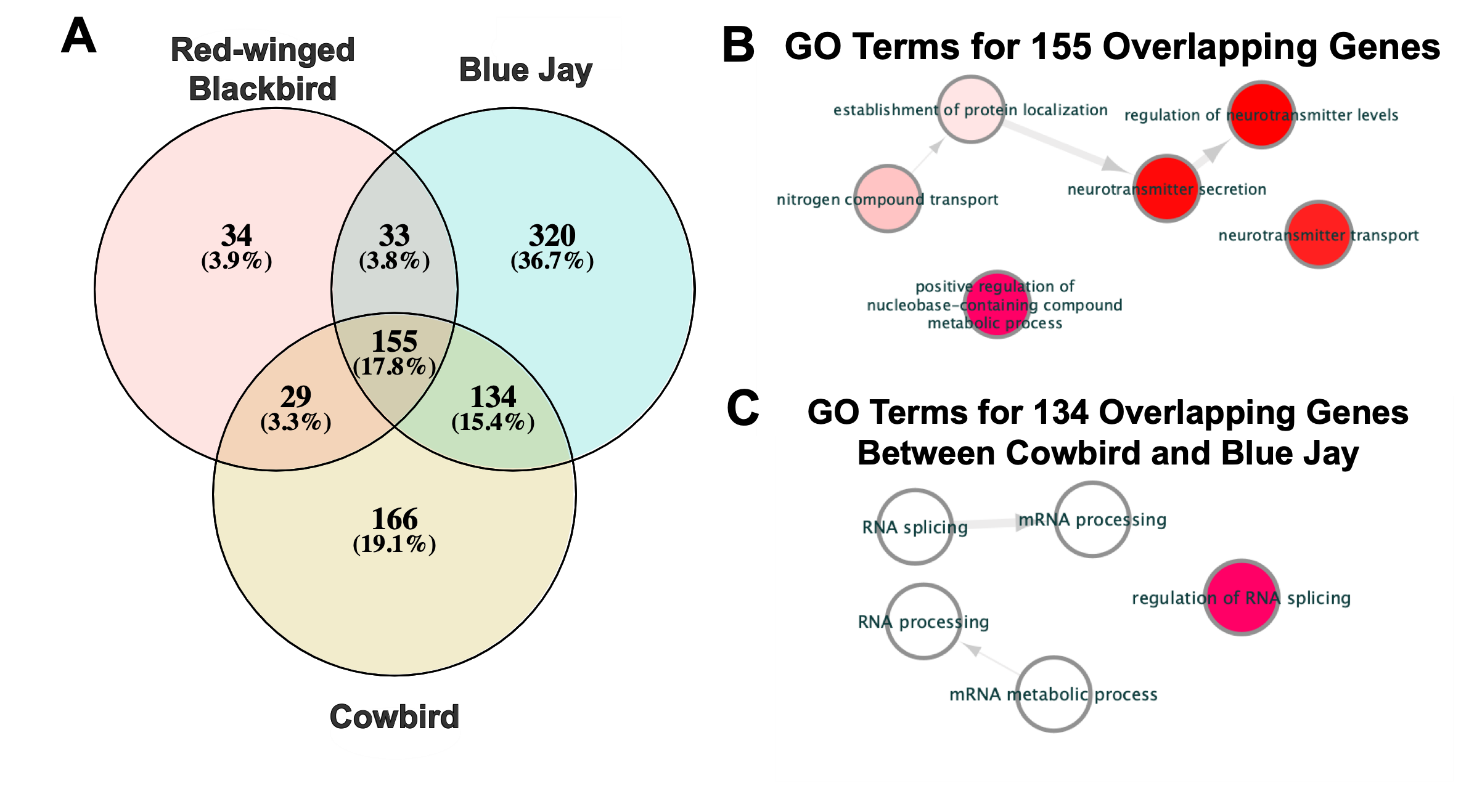


Supplementary Figure 1. Differential gene expression analysis results. A) Venn Diagram of overlapping differentially expressed genes across experimental treatments compared to the negative control (dove coo). B) GO Terms for comparisons with significant over-representation in gene ontology analysis for all three treatments and C) between cowbird and blue jay.


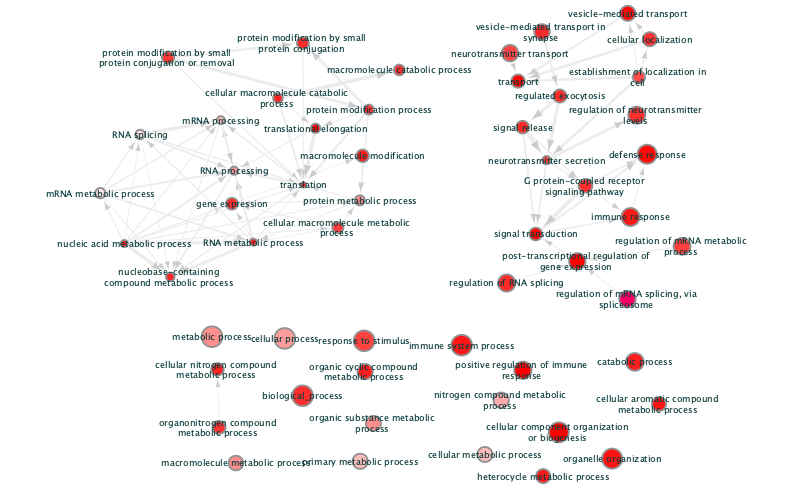


Supplementary Figure 2. Over-represented gene ontology terms from module 2 of the weighted gene coexpression network analysis.
